# The fission yeast SPB component Dms1 is required to initiate forespore membrane formation and maintain meiotic SPB components

**DOI:** 10.1371/journal.pone.0197879

**Published:** 2018-05-29

**Authors:** Touko Niimi, Taro Nakamura

**Affiliations:** Department of Biology, Graduate School of Science, Osaka City University, Sumiyoshi-ku, Osaka, Japan; Hiroshima Universtiy, JAPAN

## Abstract

The spindle pole body (SPB) plays a central role in spore plasma membrane formation in addition to its recognized role in microtubule organization. During meiosis, a biomembrane called the forespore membrane (FSM) is newly formed at the SPB. Although several SPB proteins essential for the initiation of FSM formation (meiotic SPB components) have been identified, the molecular mechanism is still unknown. Here, we report the isolation and functional characterization of Dms1 as a component of the SPB. We show that FSM formation does not initiate in *dms1Δ* cells. Dms1 protein is constitutively expressed throughout the life cycle and localizes to the SPB and the nuclear envelope. The predicted Dms1 protein has a transmembrane domain, which is required for correct localization at the SPB. Dms1 is essential for the proper localization of three meiotic SPB components, Spo15, Spo2, and Spo13, but these components do not affect localization of Dms1. Collectively, these results suggest that Dms1 anchors these meiotic SPB components to the SPB, thereby facilitating the initiation of FSM formation.

## Introduction

The fission yeast *Schizosaccharomyces pombe* cells enter sporulation under nutrient starvation, particularly when nitrogen is the limiting nutrient [1–3]. Sporulation consists of two overlapping processes, meiotic nuclear division and spore morphogenesis. The main event of the latter process is the formation of a double-layered intracellular membrane, called the forespore membrane (FSM). Formation of the FSM initiates during meiosis II. As the nucleus divides, the FSM expands by vesicle fusion and eventually encapsulates each of the four nuclei generated. Subsequently, the inner layer of the FSM becomes the spore plasma membrane, while the outer layer is degraded by autolysis [4, 5].

An interesting aspect of FSM formation is its initiation because, unlike other biological membranes, the FSM is newly synthesized within the cytoplasm of the parent cell [4–7]. FSM formation takes place at the spindle pole body (SPB), a proteinaceous structure composed of multiple layers that is equivalent to the centrosome in animal cells [8]. In *S*. *pombe*, the SPB is located in the cytoplasm close to the nuclear envelope during interphase, but becomes embedded in the nuclear envelope when cells enter meiosis [9]. During meiosis II, several meiotic outer plaques (MOPs) emerge at the cytoplasmic side of the SPB, as observed by electron microscopy [6]. This morphological change of the SPB is referred to as “SPB modification”, and has also been detected by fluorescence microscopy using anti-Sad1 antibody as a change from a compact dot to a crescent form [10]. Although it has not been shown conclusively that the morphological changes observed by electron microscopy correspond to those seen by fluorescence microscopy, modification of the SPB is presumed to be indispensable for spore formation.

Isolation and characterization of sporulation-related genes (*spo*^*+*^) have begun to unveil the molecular mechanism underlying the initiation of FSM formation [11–15]. So far, seven SPB components required for FSM formation (meiotic SPB components) have been identified: Cam1, Spo2, Spo7, Spo13, Spo15, Ypt2, and Ypt3. During vegetative growth, the coiled-coil protein Spo15 is a component of the single-plaque SPB [16], and the calmodulin orthologue Cam1 is essential for its localization to the SPB [17]. The sporulation-specific proteins Spo2 and Spo13 are produced when meiosis is initiated, and are recruited to the cytoplasmic side of the SPB via Spo15. Localization of Spo13 to the SPB is dependent on Spo2 [18]. When meiosis II starts, the meiotic SPB component Spo7 localizes to the SPB independently of Spo2, Spo13, Spo15, and Cam1, and is thought to coordinate formation of the leading edge of the FSM and the initiation of FSM assembly [19]. In addition, we have recently shown that two exocytic small GTPases from the Rab family, Ypt3 and Ypt2, play an important role in FSM formation. During meiosis, these GTPases localize at the cytoplasmic side of the SPB dependent on Spo13. Because Spo13 is known as a putative GDP/GTP exchange factor for Ypt2 [20], it is possible that the Rab cascade (i.e., Ypt3-Spo13-Ypt2) may facilitate membrane fusion on the SPB, and thus the initiation of FSM formation [15, 21].

In mammalian cells, the ciliary membrane initially forms in the vicinity of the pericentrosome and then expands by membrane vesicle fusion similar to the FSM. Therefore, the mechanism of membrane formation from the centrosome and that from the SPB might be conserved throughout evolution. It is therefore interesting to determine how the SPB functions as an origin of membrane formation, in addition to its recognized role in microtubule organization. In this study, we report the isolation and functional characterization of a novel SPB component that is essential for the recruitment of meiotic SPB components. Our analysis provides mechanistic insight into the regulation of meiotic SPB components to ensure the correct initiation of FSM formation.

## Materials and methods

### Yeast strains and media

The *S*. *pombe* and plasmids used in this study are listed in Tables 1 and 2, respectively. For vegetative cultures, complete (YE) or synthetic (SD or MM+N) media were used. For sporulation, cells precultured in YE, or SD/MM+N were incubated in malt extract medium (ME) or synthetic sporulation medium (SSA/MM-N), respectively [22, 23]. Unless stated otherwise, *S*. *pombe* strains were incubated at 30°C for growth or 28°C for mating and sporulation. Synchronous meiosis was induced by a temperature shift using strains carrying the *pat1-114* allele as previously described [24].

**Table 1:** Strains used in this study.

Strain (accession no.)^a^	Genotype	Source
L968 (FY7520)^b^	*h*^*90*^	U. Leupold
JZ670 (FY7716)^b^	*h*^*-*^*/h*^*-*^ *pat1-114/pat1-114 ade6-M210/ade6-M216 leu1-32/leu1-32*	M. Yamamoto
MM72-6B (FY6848) ^b^	*h*^*90*^ *ura4-D18*	YGRC/NBRP
MS1442 (FY17726) ^b^	*h*^*+*^ *GFP-atb2-kan*^*r*^ *cut11-3mRFP-hyg*^*r*^ *sfi1-CFP-nat*^*r*^ *his2 leu1-32 ura4-D18*	[25]
TN104 (FY7273) ^b^	*h*^90^ *ade6-M210 leu1-32*	[18]
TN105 (FY7270) ^b^	*h*^*90*^ *ade6-M210 ura4-D18*	YGRC/NBRP
TN122 (FY7033) ^b^	*h*^*-*^ *pat1-114 ade6-M210 leu1-32*	YGRC/NBRP
TN123 (FY7034) ^b^	*h*^*-*^ *pat1-114 ade6-M216 leu1-32*	YGRC/NBRP
YN89 (FY12317) ^b^	*h*^*90*^ *spo15-GFP<<LEU2 leu1-32*	[26]
AI212 (FY12991) ^b^	*h*^*90*^ *GFP-cam1 ura4-D18*	YGRC/NBRP
AI259 (FY19919) ^b^	*h*^*90*^ *leu1<<spo2-GFP*	[17]
KI36 (FY25759) ^b^	*h*^*90*^ *leu1<<GFP-psy1 ade6-M210*	[15]
KI37 (FY25760) ^b^	*h*^*90*^ *ade6-M210 leu1-32 ura4-D18*	YGRC/NBRP
KI44 (FY25631) ^b^	*h*^*90*^ *ade6<<mCherry-atb2 leu1<<GFP-psy1 ura4-D18*	[15]
KI121 (FY25784) ^b^	*h*^*90*^ *leu1<<spo13-GFP ade6-M210*	[15]
KI163a	*h*^*90*^ *spo15-mCherry<<LEU2 ade6-M210 leu1-32 ura4-D18*	Lab stock
KI175 (FY25805)	*h*^*90*^ *ade6-M210*	YGRC/NBRP
KI176 (FY25806) ^b^	*h*^*90*^ *leu1-32 ura4-D18*	YGRC/NBRP
KI195	*h*^*90*^ *spo7-GFP<<LEU2 leu1-32 ade6-M210 ura4-D18*	Lab stock
Bioneer deletion set	*h*^*+*^ *geneX*::*kanMX4 ade6-M210 or M216 leu1-32 ura4-D18*	[27]
CAL6 (FY33526)	*h*^*90*^ *SPAC6C3*.*06c*::*kanMX4 leu1<<GFP-psy1 ade6-M210*	This study
CAL8 (FY33527)	*h*^*90*^ *csn2*::*kanMX4 leu1<<GFP-psy1 ade6-M210*	This study
CAL23 (FY33528)	*h*^*90*^ *tpp1*::*kanMX4 leu1<<GFP-psy1 ade6-M210*	This study
CAL27 (FY33529)	*h*^*90*^ *mug165*::*kanMX4 leu1<<GFP-psy1 ade6-M210*	This study
CAL31 (FY33530)	*h*^*90*^ *mug166*::*kanMX4 leu1<<GFP-psy1 ade6-M210*	This study
CAL39 (FY33531)	*h*^*90*^ *erp2*::*kanMX4 leu1<<GFP-psy1 ade6-M210*	This study
CAL45 (FY33532)	*h*^*90*^ *erp5*::*kanMX4 leu1<<GFP-psy1 ade6-M210*	This study
CAL50 (FY33533)	*h*^*90*^ *spe2*::*kanMX4 leu1<<GFP-psy1 ade6-M210*	This study
CAL53 (FY33534)	*h*^*90*^ *dms1*::*kanMX4 leu1<<GFP-psy1 ade6-M210*	This study
CAL56 (FY33535)	*h*^*90*^ *mug123*::*kanMX4 leu1<<GFP-psy1 ade6-M210*	This study
CAL60 (FY33536)	*h*^*90*^ *dms1*::*kanMX4 leu1<<spo2-GFP*	This study
CAL62 (FY33537)	*h*^*90*^ *dms1*::*kanMX4 leu1<<spo13-GFP*	This study
CAL67 (FY33538)	*h*^*90*^ *dms1*::*kanMX4 spo7-GFP<<LEU2 ade6-M210 leu1-32 ura4-D18*	This study
CAL68 (FY33539)	*h*^*90*^ *dms1*::*kanMX4 spo15-GFP<<LEU2 leu1-32*	This study
CAL71 (FY33540)	*h*^*90*^ *dms1*::*kanMX4 leu1<<GFP-psy1 ade6<<mCherry-atb2*	This study
CAL78 (FY33541)	*h*^*90*^ *dms1*::*kanMX4 leu1<<spo13-GFP ade6-M210*	This study
CAL80 (FY33542)	*h*^*90*^ *dms1*::*kanMX4 leu1<<GFP-dms1 ade6-M210*	This study
CAL81 (FY33543)	*h*^*90*^ *GFP-dms1 ura4-D18*	This study
CAL83 (FY33544)	*h*^*90*^ *spo15*::*ura4*^*+*^ *GFP-dms1 ura4-D18*	This study
CAL84 (FY33545)	*h*^*90*^ *GFP-dms1 ade6<<mCherry-atb2*	This study
CAL88 (FY33546)	*h*^*90*^ *GFP-dms1 spo13-mCherry<<ura4*^*+*^ *ura4-D18*	This study
CAL89 (FY33547)	*h*^*90*^ *spo2*::*ura4 GFP-dms1 ura4-D18*	This study
CAL90 (FY33548)	*h*^*90*^ *spo7*::*ura4 GFP-dms1 ura4-D18*	This study
CAL98 (FY33549)	*h*^*90*^ *GFP-dms1 cut11-3mRFP-hyg*^*r*^ *ura4-D18*	This study
CAL99 (FY33550)	*h*^*90*^ *dms1*::*kanMX4 leu1<<GFP-dms1ΔTM ade6-M210*	This study
CAL114 (FY33551)	*h*^*90*^ *ura4-D18 [pREP42(GST)]*	This study
CAL115 (FY33552)	*h*^*90*^ *ura4-D18 [pREP42(GST-dms1)]*	This study
CAL116 (FY33553)	*h*^*90*^ *cam1-22*,*117<<ura4*^*+*^ *GFP-dms1 ura4-D18*	This study
CAL117 (FY33554)	*h*^*-*^*/h*^*-*^ *pat1-114/pat1-114 GFP-dms1/GFP-dms1 ade6-M210/ade6-M216*	This study
	*leu1-32/leu1-32*	
CAL119 (FY33555)	*h*^*90*^ *GFP-dms1 spo15-mCherry<<LEU2 ade6-M216 leu1-32*	This study
CAL120 (FY33556)	*h*^*90*^ *spo13*::*ura4 GFP-dms1 ura4-D18*	This study
CAL121 (FY33557)	*h*^*90*^ *dms1*::*kanMX4 GFP-cam1 ura4-D18*	This study
CAL124 (FY33558)	*h*^*90*^ *mug168*::*kanMX4 leu1<<GFP-psy1 ade6-M210*	This study
CAL126 (FY33559)	*h*^*90*^ *dms1*::*kanMX4 leu1<<GFP-dms1TM ade6-M210*	This study
CAL129 (FY33560)	*h*^*90*^ *spe3*::*kanMX4 leu1<<GFP-psy1 ade6-M210*	This study
CAL130 (FY33561)	*h*^*90*^ *csn1*::*kanMX4 leu1<<GFP-psy1 ade6-M210*	This study
CAL168 (FY33562)	*h*^*90*^ *dms1*::*kanMX4 leu1<<GFP-dms1TM spo15-mCherry-hyg*^*r*^ *ade6-M210*	This study
CAL169 (FY33563)	*h*^*90*^ *dms1*::*kanMX4 leu1<<GFP-dms1 spo15-mCherry-hyg*^*r*^ *ade6-M210*	This study
CAL170 (FY33564)	*h*^*90*^ *dms1*::*kanMX4 leu1<<GFP-dms1ΔTM spo15-mCherry-hyg*^*r*^ *ade6-M210*	This study
CAL178 (FY33565)	*h*^*-*^*/h*^*-*^ *dms1*::*kanMX4/dms1*::*kanMX4 pat1-114/pat1-114 ade6-M210/ade6-M216*	This study
	*leu1-32/leu1-32*	
CAL180 (FY33566)	*h*^*90*^ *dms1*::*kanMX4 spo15-mCherry-hyg*^*r*^ *ade6-M210 leu1-32 ura4-D18*	This study
	[pREP41(*GFP-dms1*)]	

^a^ Accession number are NBRP ID from the Yeast Genetic Resource Center of Japan supported by the National BioResource Project (YGRC/NBRP http://yeast.nig.ac.jp/yeast/).

^b^ These strains are obtained from YGRC/NBRP.

**Table 2:** Plasmids used in this study.

Plasmid^a^	Characteristics	Source
	***S*. *pombe***	
pBR(leu1)	*leu1*^*+*^ in pBR322	[28]
pREP41	*ars*1, *LEU*2-based expression vector carrying *nmt*41 promoter	[29]
pREP42	*ars1*, *ura4*^*+*^-based expression vector carrying *nmt*41 promoter	[30]
pAL-KS	*ars1*, *LEU2-*based multicopy shuttle vector	[30]
pREP42(GST)	pREP42, Glutathione-*S*-transferase (*GST*)	[18]
pBR(leu1)(GFP-dms1)	pBR(leu1), *GFP-dms1*	This study
pBR(leu1)(GFP-dms1ΔTM)	pBR(leu1), *GFP-dms1ΔTM*	This study
pBR(leu1)(GFP-dms1TM)	pBR(leu1), *GFP-dms1TM*	This study
pREP41(GFP-dms1)	pREP41, GFP-*dms1*^*+*^	This study
pREP42(GST-dms1)	pREP42, *GST-dms1*^*+*^	This study
	***S*. *cerevisiae***	
pGAD424	2 μ origin, *LEU2*-based vector carrying an activation domain of Gal4 and	Clontech (Mountain View,
	*ADH1* promoter	CA)
pGBT9	2 μ origin, *TRP1*-based vector carrying a DNA-binding domain of Gal4 and	Clontech
	*ADH1* promoter	
pGAD424(spo15 500aaΔ)	pGAD424, *spo15 500aaΔ*	This study
pGAD424(spo15 750aaΔ)	pGAD424, *spo15 750aaΔ*	This study
pGAD424(spo15 1000aaΔ)	pGAD424, *spo15 1000aaΔ*	This study
pGAD424(spo15 1250aaΔ)	pGAD424, *spo15 1250aaΔ*	This study
pGBT9(dms1)	pGBT9, *dms1*	This study
pGBT9(dms1ΔTM)	pGBT9, *dms1ΔTM*	This study

### Fluorescence microscopy

For fluorescence microscopy, proteins were visualized by fusion to GFP, 3 X mRFP or mCherry. The nuclear chromatin region was stained with bisbenzimide H33342 fluorochrome trihydrochroride (Hoechst 33342). Living cells were observed under a fluorescence microscope (model BX51; Olympus, Tokyo, Japan), and images were obtained by a BX51 fluorescence microscope (Olympus) equipped with an ORCA-R2 camera (Hamamatsu Photonics, Hamamatsu, Japan). Filter sets U-MWU (Olympus), U-MWIB (Olympus), and U-MWIG2 (Olympus) were used for Hoechst 33342, GFP and mCherry/3 X mRFP, respectively. Image acquisition and processing were carried out by using Aquacosmos (Hamamatsu Photonics) and Image J (National Institutes of Health, Bethesda, USA) software. Time-lapse observation was performed as follows. Cells were incubated on SSA at 28°C for 16 hours. After conjugation, cells were inoculated to SSA medium on a cell-culture dish with a glass bottom (Greiner Bio-One, Frickenhausen, Germany) and observed under a fluorescence microscope (model IX71; Olympus) as described in Nakamura et al. (2008) [31]. Images were processed with Image J.

### Western blotting

Proteins were resolved by SDS–PAGE and then transferred to a polyvinylidene difluoride membrane (Immobilon-P; Merck Millipore, Darmstadt, Germany). Blots were probed with a rabbit (Medical and Biological Laboratories, Nagoya, Japan) or rat anti-GFP antibody (a gift from S. Fujita, Mitsubishi Kagaku Institute of Life Sciences), and the anti-α-tubulin antibody TAT-1 [32]. Immunoreactive bands were revealed by ECL select chemiluminescence (GE Healthcare, Buckinghamshire, UK) with horseradish peroxidase-conjugated goat anti-rabbit IgG (Bio-Rad Laboratories, Hercules, USA) or goat anti-mouse IgG (BIOSOURCE, Camarillo, USA).

## Results

### The novel gene *dms1*^*+*^ is involved in FSM formation

**Fig 1:**
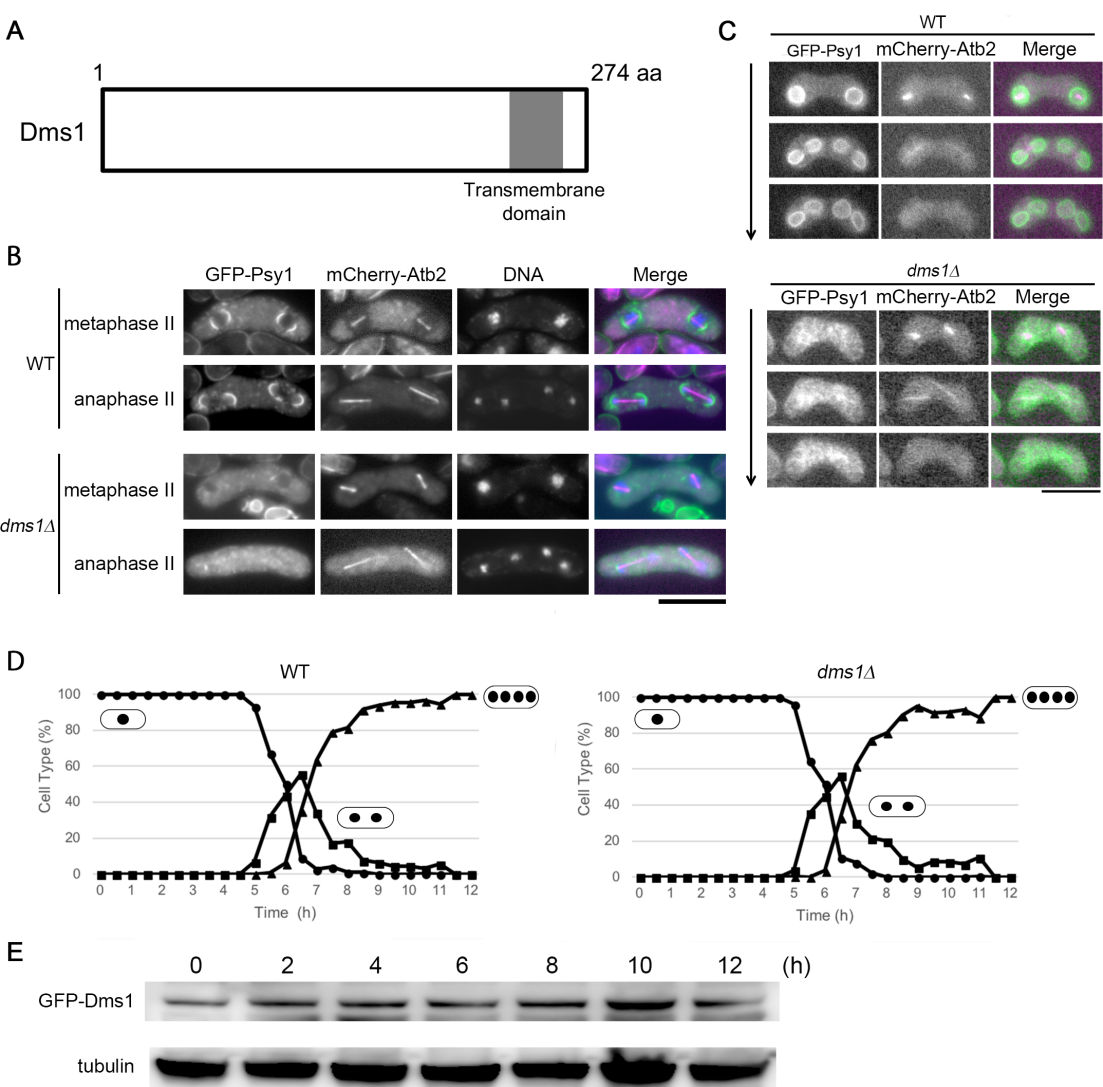
Dms1 is essential for the initiation of FSM formation. (A) Schematic diagram of Dms1. The gray box indicates the putative transmembrane region. (B) FSM formation in the *dms1Δ* mutant. Wild-type (KI44) and *dms1Δ* (CAL71) cells expressing GFP-Psy1 and mCherry-Atb2, a microtubule marker, were sporulated on MEA medium for 16 hours. Chromosomal DNA was stained with Hoechst 33342 and analyzed by fluorescence microscopy. GFP-Psy1 (green), mCherry-Atb2 (magenta), and Hoechst 33342 (blue) are overlaid in the merged images. Bar, 10 μm. (C) FSM formation in living *dms1Δ* cells. Wild-type (KI44) and *dms1Δ* (CAL71) cells expressing GFP-Psy1 and mCherry-Atb2 were analyzed by time-lapse analysis. GFP-Psy1 (green) and mCherry-Atb2 (magenta) are overlaid in merged images. Bar, 10 μm. (D) Homozygous diploid *pat1* (JZ670) and *pat1dms1Δ* (CAL178) cells were cultured to mid log phase, transferred to MM-N medium for 16 hours at 25°C, and then shifted to 34°C to inactivate Pat1 and synchronize meiosis. Progression of meiosis was monitored by DAPI staining of samples that were collected every 30 min after the temperature shift. At least 200 cells were scored by fluorescence microscopy at each time point. Circle, mononucleate; square, binucleate; triangle, tri- or tetranucleate cells. (E) Expression level of Dms1 during meiosis. A homothallic haploid wild-type strain expressing GFP-Dms1 (CAL81) was incubated in MM-N, aliquots were removed every 2 hours, and the protein extract was subjected to western blot analysis with a rabbit anti-GFP antibody and a mouse anti-α-tubulin antibody as a loading control.

Genome-wide reverse genetic studies have shown that many *S*. *pombe* genes are involved in sporulation [33, 34]. Among them, we selected 13 deletion mutants that have been reported to show defects in sporulation but have not been uncharacterized (*mug123*, *mug165*, *mug166*, *mug168*, *csn1*, *csn2*, *erp2*, *erp5*, *spe2*, *spe3*, *tpp1*, *SPAC6C3*.*06c*, and *SPCC1739*.*04c)*. We introduced GFP-tagged Psy1, an FSM marker, into each mutant and observed formation of the FSM by fluorescence microscopy (S1 Fig). Because *SPCC1739*.*04cΔ* showed the most marked defect in FSM formation (S1 Fig), we characterized it further. Recently, Blyth *et al*. (2017) reported a functional genomics screen of genes important in *S*. *pombe* meiosis in which they identified *SPCC1739*.*04c* as *dms1* [35]. Therefore, *SPCC1739*.*04c* is designated as *dms1*^*+*^ hereafter. The *dms1*^*+*^ gene was predicted to encode a 31-kDa protein composed of 274 amino acids. The deduced amino acid sequence was found to be conserved only in the *Schizosaccharomyces* genus. Hydropathic profiling and prediction of secondary structure indicated that Dms1 contains one potential membrane-spanning domain in its carboxy terminus (Fig 1A), but no other known functional motifs.

### Dms1 is essential for initiating FSM formation

To examine how the *dms1* mutation impairs sporulation, assembly of the FSM in the *dms1* mutant was observed in detail by using GFP-Psy1. In wild-type cells at metaphase II, the GFP-Psy1 signal was observed as two cup-like structures at each end of the spindle microtubules, which were visualized by an mCherry-labeled α-tubulin, Atb2 (Fig 1B). In contrast, no GFP-Psy1 signal was observed at the spindle poles in about 90% of *dms1Δ* cells at the same stage (Fig 1B), a result that was confirmed by live-cell imaging (Fig 1C; S1 and S2 Movies). This phenotype is similar to that of mutants of meiotic SPB components such as Spo13 and Spo15 [16, 18]. Taken together, these data indicate that Dms1 is essential for the initiation of FSM formation.

In complete medium, the *dms1* deletion mutant did not differ from the wild-type strain in growth rate, cell size, or shape (data not shown), indicating that *dms1*^*+*^ is not essential for vegetative growth. Next, we examined the progression of meiotic nuclear division in *dms1Δ* cells. To synchronize meiosis effectively, we used the *pat1-114* temperature-sensitive strain, which enters meiosis in a highly synchronous manner when it is shifted to its restrictive temperature, 34°C [24]. The first and second meiotic divisions of the *pat1dms1Δ* cells were found to proceed with kinetics similar to that observed for *pat1* cells, with a final yield of tetranucleate cells of 90% (Fig 1D). Therefore, the *dms1Δ* mutant seems to undergo meiosis normally, but is defective in ascospore formation.

### Dms1 localizes at the SPB and the nuclear envelope

A comprehensive mRNA expression study previously revealed that the *dms1*^*+*^ gene is expressed throughout the life cycle, but is upregulated slightly during meiosis and/or sporulation [36]; however, the levels of Dms1 protein during meiosis/sporulation have not been reported. To ascertain whether the levels of Dms1 protein mirror those of *dms1* mRNA, we constructed a strain in which GFP-tagged *dms1*^*+*^ was expressed from its endogenous locus. As expected, GFP-Dms1 was detected during vegetative growth and its abundance essentially remained constant after the induction of sporulation (Fig 1E).

**Fig 2:**
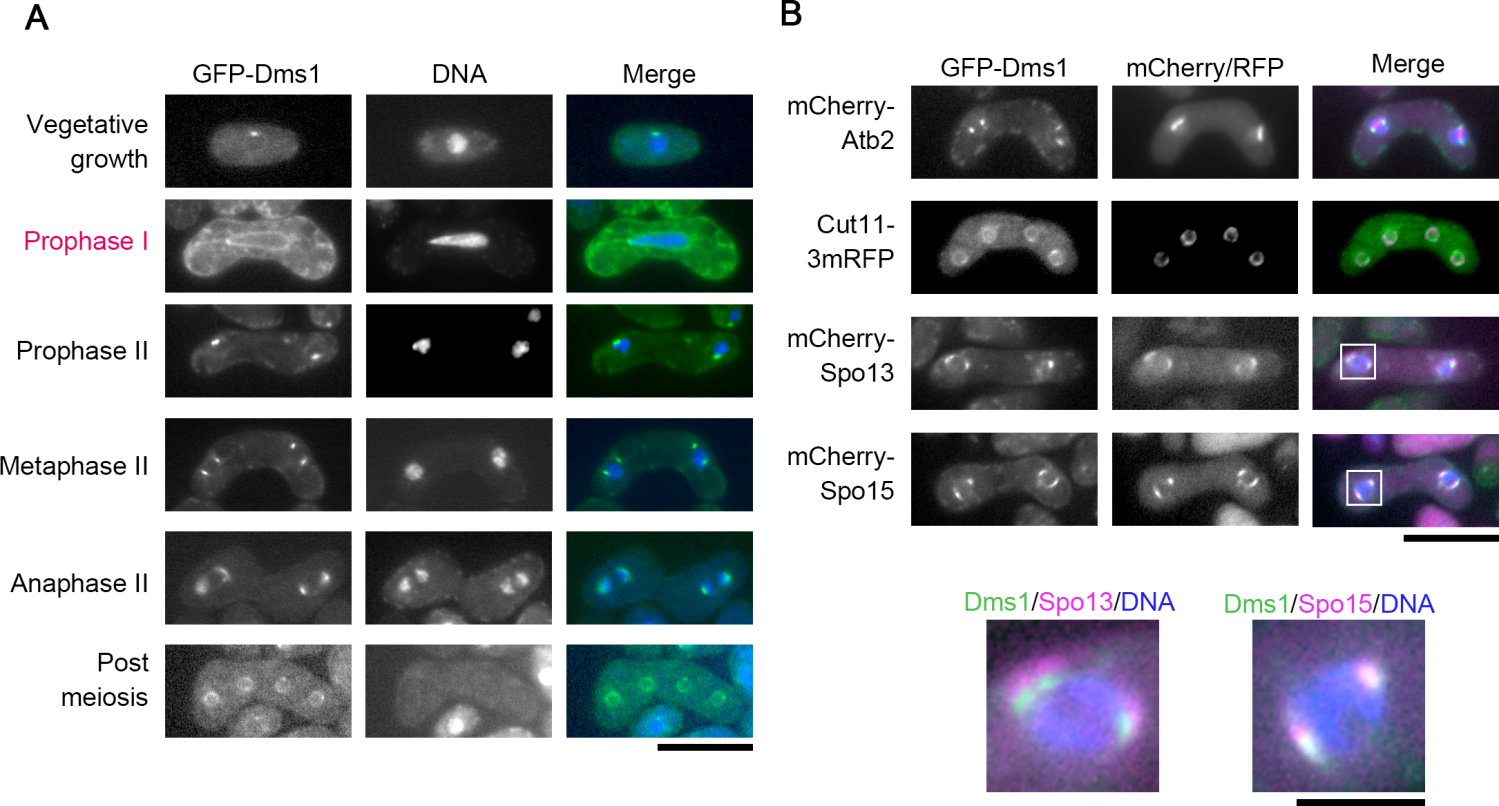
Dms1 localizes at the SPB and the nuclear envelope. (A) Localization of GFP-Dms1 during vegetative growth and sporulation. A homothallic haploid wild-type strain expressing GFP-Dms1 (CAL81) was grown on YEA for 1 day or was sporulated on MEA for 16 hours. Chromosomal DNA was stained by Hoechst 33342 and analyzed by fluorescence microscopy. GFP-Dms1 (green) and Hoechst 33342 (blue) are overlaid in the merged images. Bar, 10 μm. (B) Dual observation of Dms1 and microtubules, the nuclear envelope, or meiotic SPB components. Homothallic haploid wild-type cells expressing GFP-Dms1 and Cut11-3mRFP (CAL98), mCherry-Atb2 (CAL84), Spo13-mCherry (CAL88), or Spo15-mCherry (CAL119) were sporulated on MEA for 16 hours. GFP-Dms1 (green), Cut11-3mRFP, mCherry-Atb2, Spo13-mCherry, or Spo15-mCherry (magenta) and Hoechst 33342 (blue) are overlaid in the merged images. Bar, 10 μm. High magnification-images of the regions in the white squares are shown below. Bar, 1 μm.

The subcellular localization of Dms1 was observed by fluorescence microscopy. Consistent with the western blot analysis, GFP-Dms1 signal was observed in both vegetative and in meiotic cells as one or two dots in the periphery of nuclei (Fig 2A). These dots were detected at both ends of the spindle microtubules at meiosis II (Fig 2B), indicating that Dms1 localizes to the SPB. In addition, a weak signal surrounding the nucleus was also observed in vegetative cells. This signal became stronger after meiosis II completed and overlapped with signals from RFP-tagged Cut11 (Fig 2B), a marker of the nuclear envelope [37]. We therefore concluded that Dms1 is also localized to the nuclear envelope.

**Fig 3:**
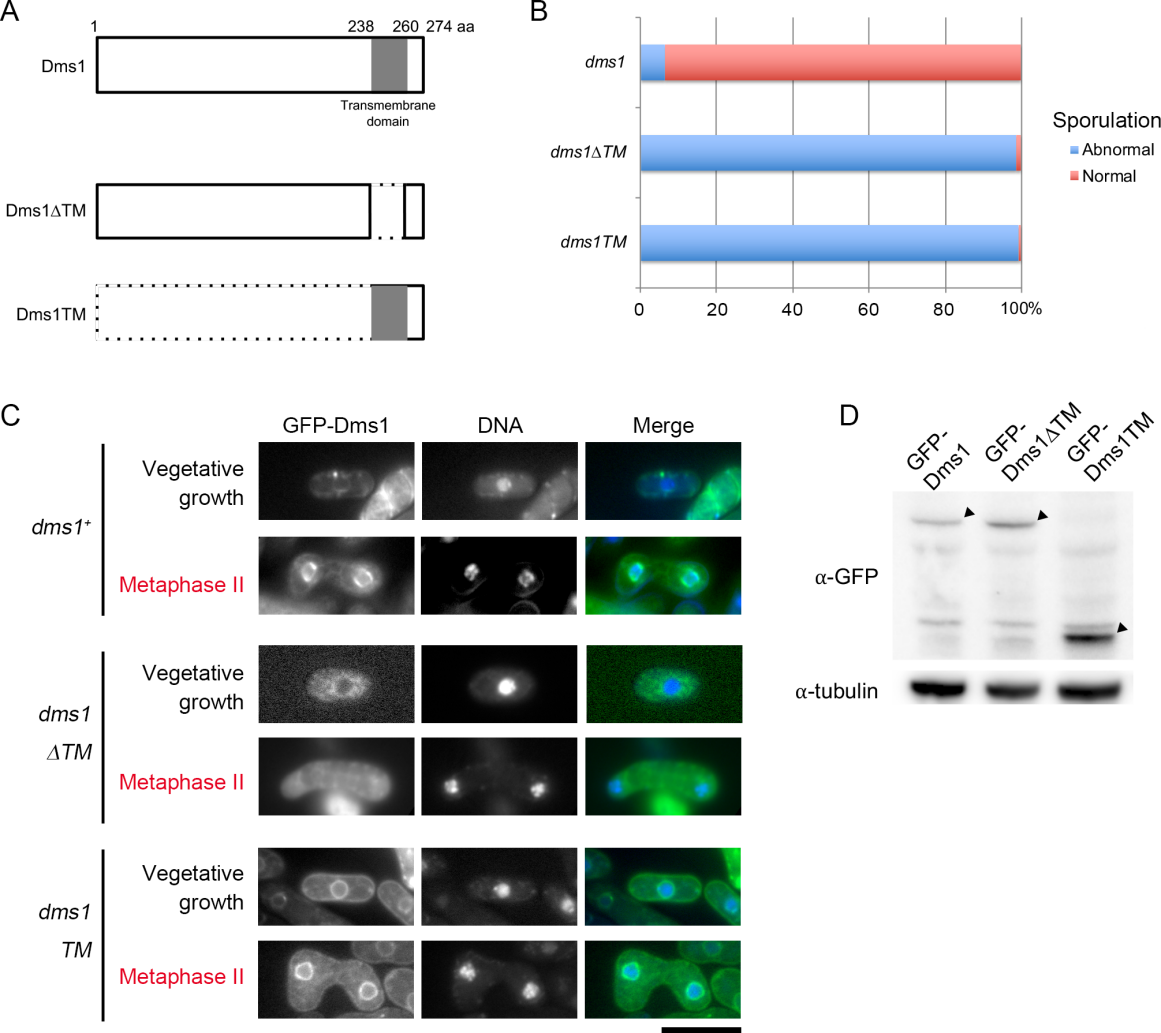
The transmembrane region is essential for proper localization of Dms1. (A) Schematic diagram of the *dms1* truncated mutants. The gray box indicates the putative transmembrane domain. (B) Sporulation rate of *dms1* mutants. A homothallic haploid *dms1Δ* strain expressing GFP-Dms1 (CAL80), GFP-Dms1ΔTM (CAL99), or GFP-Dms1TM (CAL126) was sporulated on MEA for 2 days. (C) Localization of Dms1 mutant proteins. A homothallic haploid *dms1Δ* strain expressing GFP-Dms1 (CAL80), GFP-Dms1ΔTM (CAL99), or GFP-Dms1TM (CAL126) was grown on YEA for 1 day or was sporulated on MEA for 16 hours. Chromosomal DNA was stained by Hoechst 33342 and analyzed by fluorescence microscopy. GFP-Dms1, GFP-Dms1ΔTM, or GFP-Dms1TM (green) and Hoechst 33342 (blue) are overlaid in the merged images. Bar, 10 μm. (D) Detection of the Dms1 mutant proteins by western blotting. Homothallic haploid *dms1Δ* cells expressing GFP-Dms1 (CAL80), GFP-Dms1ΔTM (CAL99), and GFP-Dms1TM (CAL126) were cultured in MM+N. Protein extracts were subjected to western blot analysis with mouse anti-GFP and anti-α-tubulin antibody as a loading control. Arrowheads indicate Dms1 and truncated-Dms1 mutant proteins.

As described above, the predicted Dms1 protein has a transmembrane region. To assess the role of this transmembrane domain, we constructed a *dms1ΔTM* mutant lacking this domain (Fig 3A). Spore formation was severely compromised in the *dms1ΔTM* mutant (Fig 3B). Consistent with this, GFP-Dms1ΔTM was not observed at the SPB but was dispersed throughout the cytoplasm (Fig 3C). The levels of GFP-Dms1ΔTM protein were comparable to those of wild-type GFP-Dms1 (Fig 3D), demonstrating that the sporulation defect of *dms1ΔTM* was not due to degradation of the mutant protein.

To determine whether the transmembrane domain alone is sufficient for the localization of Dms1, we expressed the GFP-fused transmembrane domain of Dms1 in the *dms1Δ* strain (*dms1TM*). As shown in Fig 3C, although GFP signal was clearly detected at the periphery of the nucleus and partially at the SPB, the *dms1TM* mutant did not undergo sporulation (Fig 3B). Thus, these data suggest that, via its transmembrane domain, Dms1 localizes at the nuclear envelope and the SPB, where it carries out its sporulation function.

### Dms1 is essential for cellular localization of Spo15, Spo2, and Spo13

To examine the relationship between Dms1 and other meiotic SPB components, we simultaneously expressed GFP-Dms1 and either Spo13-mCherry or Spo15-mCherry. As shown in Fig 2B, GFP-Dms1 was in close contact with both proteins at the SPB. Interestingly, the GFP-Dms1 signal was positioned within the Spo13-mCherry signal at the SPB, but overlapped with the Spo15-GFP signal.

**Fig 4:**
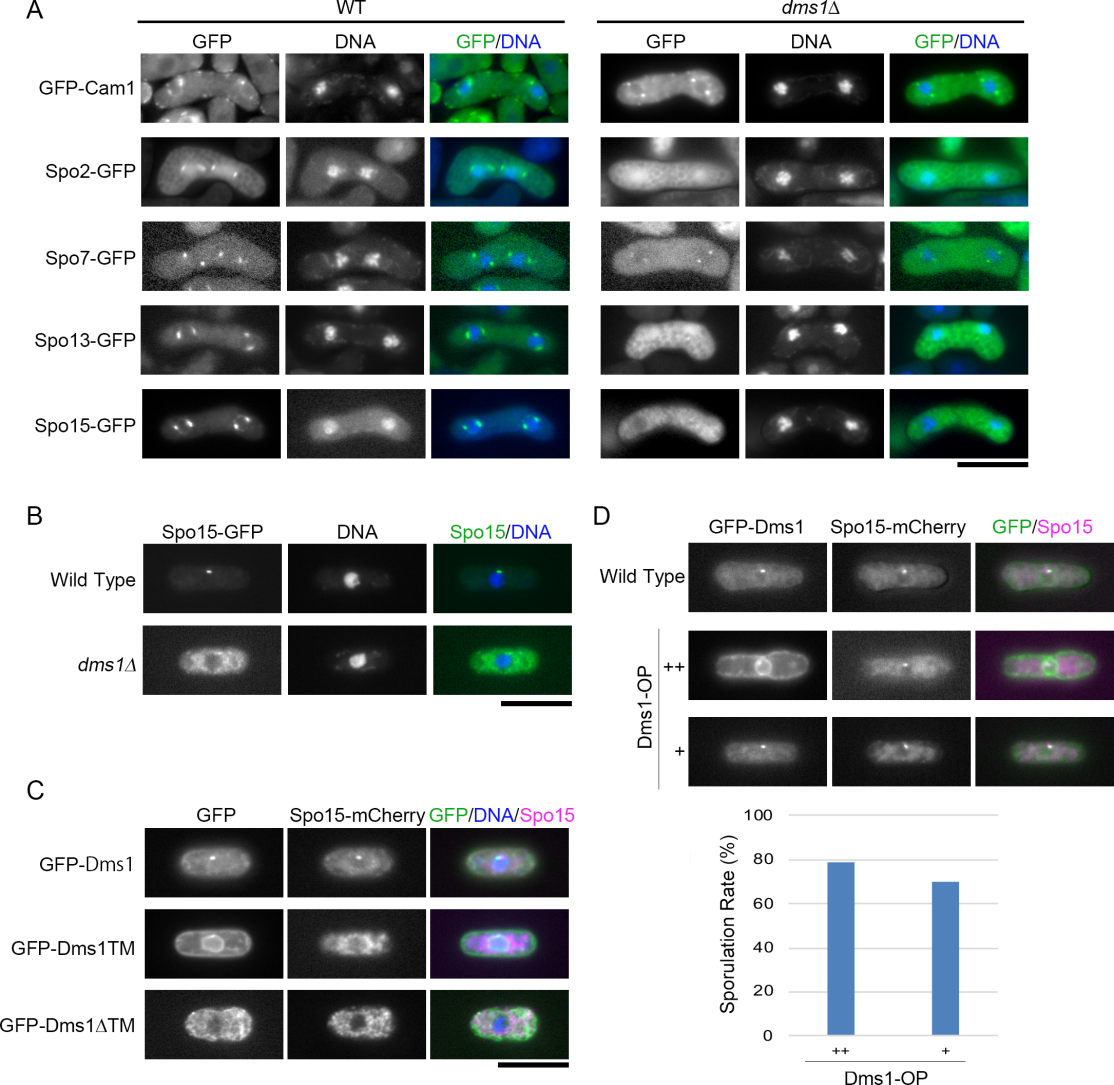
Dms1 is essential for the localization of Spo15, Spo2, and Spo13 at the SPB. (A) Localization of SPB components in *dms1Δ*. Homothallic haploid wild-type strain (left panel) expressing GFP-Cam1 (AI212), Spo2-GFP (AI259) Spo7-GFP (KI195), Spo13-GFP (KI121) or Spo15-GFP (YN89) and *dms1Δ* strain (right panel) expressing GFP-Cam1 (CAL121), Spo2-GFP (CAL60), Spo7-GFP (CAL67), Spo13-GFP (CAL78) or Spo15-GFP (CAL68) were sporulated on MEA for 16 hours. Chromosomal DNA was stained by Hoechst 33342 and analyzed by fluorescence microscopy. GFP (green) and Hoechst 33342 (blue) are overlaid in the merged images. (B) Localization of Spo15 during vegetative growth. Homothallic wild-type (YN89) and *dms1Δ* strain (CAL68) strains expressing Spo15-GFP were grown on YEA for 1 day. Chromosomal DNA was stained by Hoechst 33342 and analyzed by fluorescence microscopy. GFP (green) and Hoechst 33342 (blue) are overlaid in the merged images. (C) Homothallic haploid *dms1Δ* strains expressing Spo15-mCherry and GFP-Dms1 (CAL169), GFP-Dms1ΔTM (CAL170), or GFP-Dms1TM (CAL168) were grown on YEA. Chromosomal DNA was stained by Hoechst 33342 and analyzed by fluorescence microscopy. GFP (green), Spo15-mCherry (magenta) and Hoechst 33342 (blue) are overlaid in the merged images. (D) Homothallic haploid strains CAL169 and CAL180 expressing GFP-Dms1 and Spo15-mCherry were incubated on SSA in the presence (+) and the absence (++) of thiamine. GFP (green) and Spo15-mCherry (magenta) are overlaid in the merged image. The sporulation efficiency of these strains is also shown. Bars. 10 μm.

**Fig 5:**
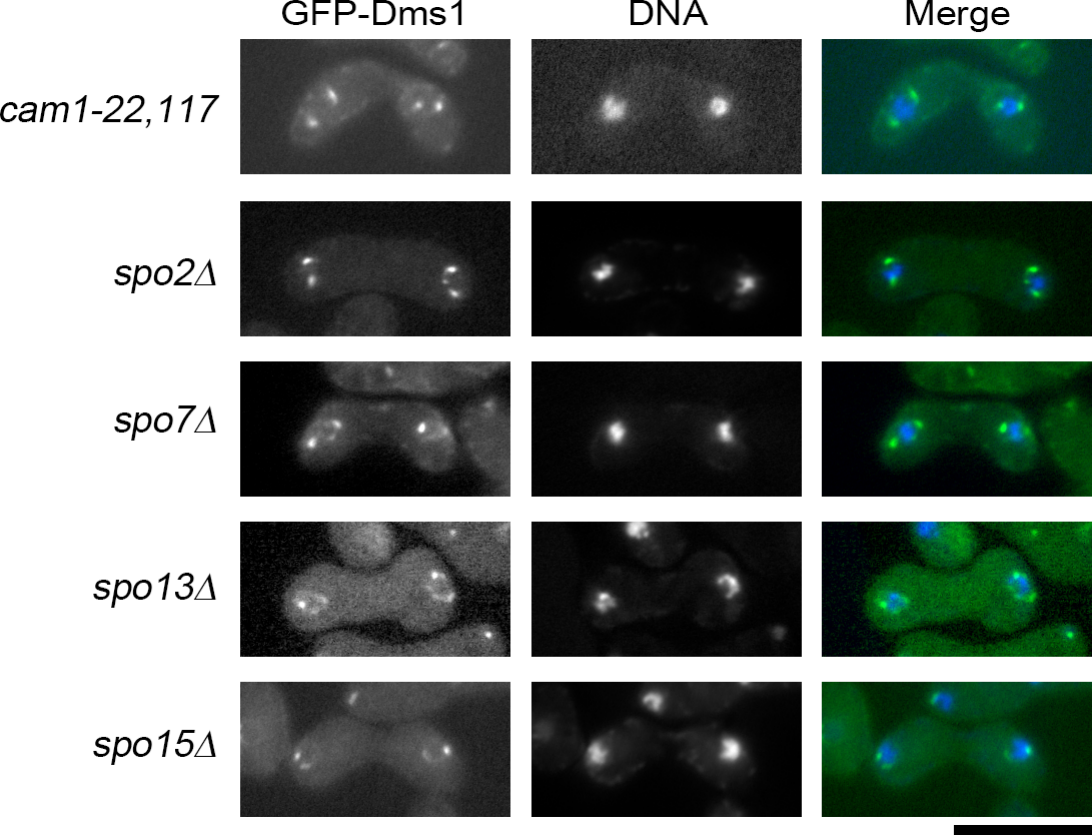
Dms1 localizes at the SPB in mutants of other meiotic SPB components. Homothallic haploid *cam1-22*,*117* (CAL116), *spo2Δ* (CAL89), *spo7Δ* (CAL90), *spo13Δ* (CAL120), and *spo15Δ* (CAL83) cells expressing GFP-Dms1 were sporulated on MEA for 16 hours. Chromosomal DNA was stained by Hoechst 33342 and analyzed by fluorescence microscopy. GFP (green) and Hoechst 33342 (blue) are overlaid in the merged images. Bar, 10 μm.

Recruitment of Spo2, Spo13, and Spo15 to the SPB is known to be strictly controlled: localization of Spo13 depends on both Spo15 and Spo2, whereas that of Spo2 depends on only Spo15 [18]. We therefore examined whether the *dms1Δ* mutation affects the localization of these proteins. In wild-type vegetative cells, Spo15-GFP localized to the SPB as previously reported [16]. By contrast, the Spo15-GFP signal was not restricted to the SPB but was diffused throughout the cytoplasm in *dms1Δ* cells during both vegetative growth and sporulation (Fig 4A and 4B), indicating that the localization of Spo15 to the SPB requires Dms1 function. In addition, Spo2-GFP and Spo13-GFP signals were not detected at the SPB, but were dispersed in the nucleus in *dms1Δ* cells (Fig 4A). These observations are similar to our previous finding that Spo2 and Spo13 localize in the nucleus in the *spo15* deletion strain [18]. By contrast, GFP-Dms1 localized to the SPB in *spo2Δ*, *spo13Δ*, and *spo15Δ* cells (Fig 5). Moreover, Spo15-GFP did not localize at the SPB in *dms1ΔTM* or *dms1TM* cells (Fig 4C), supporting our findings that the transmembrane region of Dms1 is essential for its localization and function. As described above, Dms1 also localizes to the nuclear envelope (Fig 2A). The signal at the nuclear envelope became stronger when Dms1 was overexpressed (Fig 4D). Moreover, GFP-Spo45 signal was also observed at the cell periphery. Even in these cells, the distribution of Spo15-mCherry signals was essentially identical to that of wild strain (Fig 4D). Consistent with these results, Dms1 overexpression did not affect the sporulation frequency (Fig 4D). Taken together, these data suggest the possibility that Dms1 anchors Spo15 via other SPB component(s), and thereby Spo2 and Spo13, at the SPB. To further examine this possibility, we constructed a plasmid for expressing a Spo15-GFP-Dms1TM fusion protein, in which Spo15 was fused to the transmembrane region of Dms1, and introduced it into the *dms1Δ* strain. However, Spo15-GFP-Dms1TM signal was not detected at the SPB and the fusion gene did not suppress the sporulation defect of *dms1Δ* cells. Western analysis revealed that the amount of the fusion protein was remarkably decreased compared to the wild-type Spo15 (data not shown). Therefore, we presume that the defect of suppression by Spo15-GFP-Dms1TM is due to degradation of the mutant protein.

Previously, we showed that the calmodulin Cam1 regulates the initiation of FSM formation by recruiting the Spo15-Spo2-Spo13 complex to the SPB [17]. Next, therefore, we examined the behavior of Cam1 in *dms1Δ* cells. GFP-Cam1 signal was detected at the SPB in *dms1Δ* cells as in wild-type cells. Reciprocally, GFP-Dms1 was localized at the SPB in *cam1* mutant cells (Figs 4A and 5), suggesting that Dms1 does not interact with Cam1.

Lastly, we examined the meiotic SPB component Spo7, which is known to localize to the SPB independently of Spo2, Spo13, Spo15, and Cam1 [19]. Similar to Cam1, localization of Spo7 at the SPB was not affected by the *dms1Δ* mutation (Fig 4A), and GFP-Dms1 was localized at the SPB in *spo7* mutant cells (Fig 5).

## Discussion

In *S*. *pombe*, the SPB functions as an origin of FSM formation. Although several meiotic SPB components have been identified, the full picture of how these proteins are recruited to the SPB remains unclear. In this study, we have examined the SPB component Dms1, showing that it has a transmembrane domain and locates at the innermost position of known meiotic components on the SPB. Our findings suggest the possibility that Dms1 anchors other meiotic SPB components to the SPB, thereby regulating the initiation of FSM formation.

Although FSM formation initiates in meiosis, Dms1 is expressed even in vegetative cells, as revealed previously by DNA microarray [36] and here in western blot analyses (Figs 1 and 2). Fluorescence microscopy also showed that GFP-Dms1 localizes at the SPB throughout the mitotic cell cycles and meiotic nuclear divisions, confirming the constitutive expression of *dms1*^*+*^. The structure and behavior of SPBs and spindles appeared normal in *dms1Δ* cells, consistent with the fact that *dms1* null mutants exhibited no apparent defects in either mitosis or meiotic nuclear division. Interestingly, expression of *dms1*^*+*^ was previously found to be upregulated under various environmental stresses such as heat shock, heavy metal, oxygen, and osmotic shock [36], suggesting the possibility that Dms1 is involved in the stress response. We found, however, that the growth of *dms1Δ* cells under these stresses was indistinguishable from that of wild-type cells (S2 Fig). The possibility that Dms1 plays a dispensable role in mitotically growing cells also remains to be tested.

In *Saccharomyces cerevisiae*, sporulation proceeds in a similar manner to that in *S*. *pombe* [38]. Assembly of the prospore membrane, corresponding to the *S*. *pombe* FSM, initiates with modification of the SPB during meiosis II. The prospore membrane elongates by membrane vesicle fusion, and encapsulates the haploid nucleus generated by two rounds of meiotic nuclear division. Despite these similarities, the structure of known meiotic SPB components are poorly conserved between *S*. *cerevisiae* and *S*. *pombe*. Consistent with this, to our knowledge, no meiotic SPB component with a transmembrane domain has been identified in *S*. *cerevisiae*. Duplication of the SPB also occurs slightly differently in these two yeasts: the *S*. *cerevisiae* SPB remains embedded in the nuclear membrane throughout the cell cycle, whereas the *S*. *pombe* SPB spends most of interphase in the cytoplasm adjacent to the nuclear envelope and duplicates there. After duplication, the *S*. *pombe* SPB is associated with the cytoplasmic face of the nuclear envelope. As the cell enters M phase, the nuclear envelope becomes fenestrated and the SPBs separate and enter the nucleus, forming the spindle microtubule [9, 39, 40]. We therefore presume that structural differences in the SPB components of the two yeasts may be due to the behavior of the SPB.

As mentioned above, Blyth et al. recently reported that Dms1 has a role in sporulation [35]. Via an immunoprecipitation assay and mass spectrometry, they showed that Dms1 interacts with Spo15; therefore, they suggested that Dms1 has a role in FSM formation via Spo15, although they did not examine this experimentally. To confirm whether Dms1 binds to Spo15 directly, we performed a yeast two-hybrid assay and pull-down assay. However, no positive interaction between these two proteins was detected in either assay (S3 Fig), suggesting that Dms1 may bind to Spo15 via unknown protein. Blyth *et al*, also reported that *dms1Δ* cells show abnormal behavior of spindle microtubules in meiosis II and suggested that Dms1 regulates the spacing of nuclei produced by meiosis [35]. We consider that the defect of sporulation in *dms1Δ* cells is mainly due to a fault in the recruitment of SPB components including Spo15, Spo2, and Spo13 which are essential for initiation of FSM formation. In addition, we did not observe a defect of the meiotic nuclear division in *dms1Δ* cells (Fig 1D); however, because both spindle formation and FSM formation initiate at the SPB, it is possible that Dms1 coordinates the progression of meiotic nuclear division and FSM formation.

**Fig 6:**
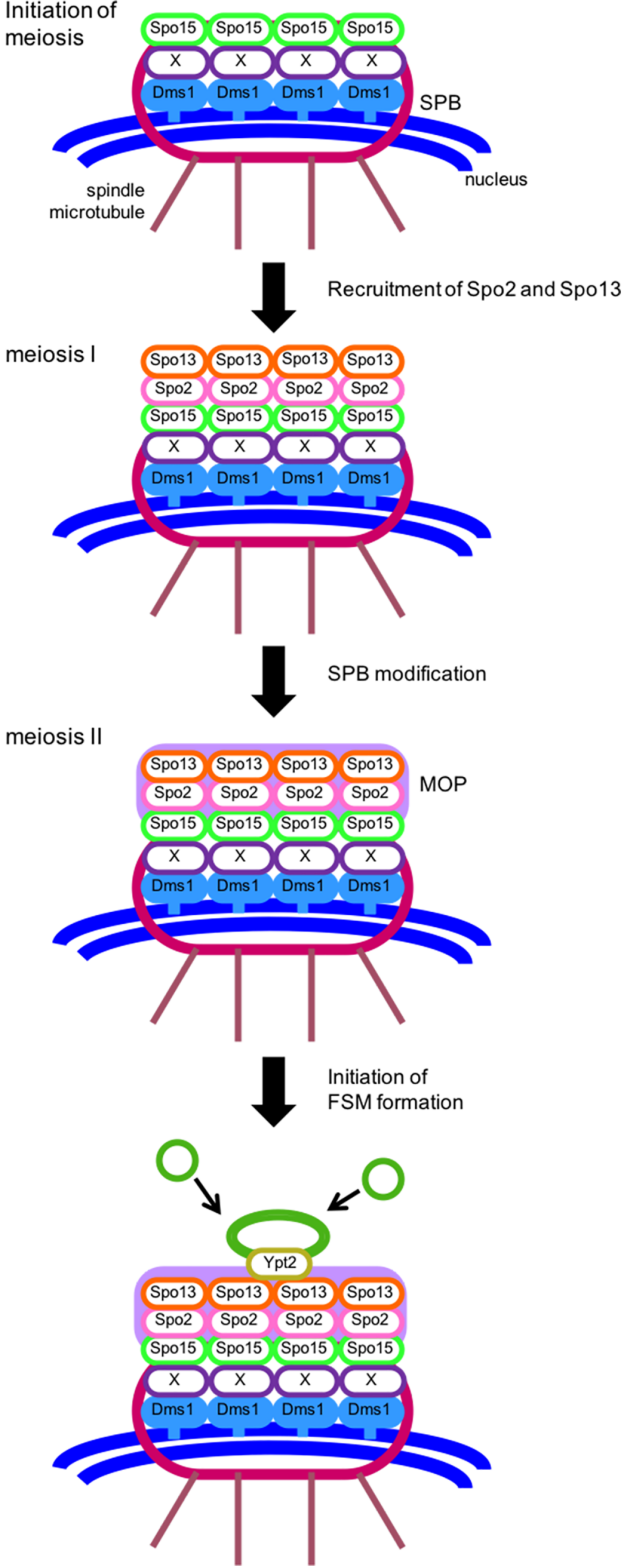
Model of the role of Dms1 in the initiation of FSM formation.

Fig 6 illustrates our working hypothesis in which Dms1 is involved in construction of the meiotic SPB. During vegetative growth, Dms1 localizes to the SPB through its transmembrane domain. Dms1 also shows a hierarchical arrangement, localizing at the most nuclear side of the SPB among the known meiotic SPB components (i.e., Cam1, Spo2, Spo7, Spo13, Spo15, Ypt2, and Ypt3). Subsequently, Spo15 is recruited to the SPB and may bind to Dms1 via an unknown protein, X. Our previous study showed that the calmodulin Cam1 binds to Spo15, and this interaction is essential for SPB localization of Spo15. However, Dms1 and Cam1 localize to the SPB independently (Fig 4A and 4B). Consistent with this, no interaction was detected between Dms1 and Cam1 by a yeast two-hybrid or pull-down assay (data not shown). During meiosis I, Spo2 and Spo13 are expressed and localized to the SPB, constructing the MOP. Spo13 then recruits and anchors the Rab-family GTPase Ypt2 [15]. When cells proceed to meiosis II, the MOP is differentiated (“SPB modification”) by a currently unknown mechanism, and the FSM, which is continuously associated with the MOP, expands by membrane vesicle fusion.

How is Dms1 connected to the SPB? The transmembrane region of Dms1 is sufficient for its localization at the nuclear envelope but not at the SPB (Fig 3C). One possibility is that the N-terminal region of Dms1 interacts with core components of the SPB and stabilizes its localization. Several SPB components have been identified. We examined the physical interaction of Dms1 with seven of them (Sad1, Pcp1, Cdc11, Sid4, Ppc89, Kms1 and Cut11), but did not obtain positive results (data not shown). A comprehensive analysis of Dms1 interaction with SPB components will be necessary in future studies.

As described above, FSM formation shares similarities with mammalian primary ciliogenesis, in which the plasma membrane is newly synthesized in the vicinity of the centrosome. The distal appendages (DAPs) of centrioles are thought to anchor cilia to the plasma membrane, suggesting that DAPs might play a role similar to the MOP. Recently, several DAP components have been identified. Similar to meiotic SPB components, they localize to the DAP in a hierarchical manner that is essential for ciliogenesis [41, 42]. However, these DAP components share very low structural similarity to those of the *S*. *pombe* MOP. On the other hand, Rab family proteins and their regulators are involved in both formation of the ciliary membrane and formation of the FSM [15, 43–46]. Thus, the mechanism by which the MOP (DAP) activates Rab proteins, thereby initiating membrane formation, should be elucidated in future studies.

In conclusion, we have identified and characterized Dms1 as a SPB component that is crucial for FSM formation, and shown that it maintains meiotic SPB components at the SPB, thereby regulating the initiation of FSM formation. Our findings will help to better understand not only the molecular mechanisms of FSM formation but also the function of the SPB as an origin of membrane organization.

## Supporting information

S1 Text Pull-down assay. (DOCX)

S1 Fig FSM formation in various mutants. Homothallic haploid wild-type (KI36), *csn1Δ* (CAL130), *csn2Δ* (CAL8), *erp2Δ* (CAL39), *erp5Δ* (CAL45), *mug123Δ* (CAL56), *mug165Δ* (CAL27), *mug166Δ* (CAL31), *mug168Δ* (CAL124), *SPAC6C3*.*06cΔ* (CAL6), *spe2Δ* (CAL50), *spe3Δ* (CAL129), *dms1Δ* (CAL53), and *tpp1Δ* (CAL23) cells expressing GFP-Psy1 were sporulated on MEA for 16 hours and analyzed by fluorescence microscopy. Bar, 10 μm.
(TIF)

S2 Fig *dms1Δ* is not sensitive to various stress. Homothallic haploid wild-type (L968, KI175, and TN105) and *dms1Δ* (CAL72, CAL92, and CAL93) strains were serially diluted from 10^2^ to 10^7^ cells/ml, spotted onto YEA, YEA + 1mM H_2_O_2_, YEA + 40 μM CdSO_4_ or YEA + 1.2 M sorbitol plates, and incubated at 30°C for 2 days. To test heat shock stress, cells were spotted onto YEA and treated at 50°C for 30 min before incubation at 30°C for 2 days.
(TIF)

S3 Fig Physical interaction of Dms1 with Spo15. (A) Yeast two-hybrid analysis. Plasmids expressing the respective Gal4 activation domain (AD) and Gal4 DNA-binding domain (BD) fusions were tested for two-hybrid interaction. (B) Pull-down assay. Cell extracts were prepared from vegetative cells expressing the tagged proteins, GST or GST-Dms1, were subjected to pull down with anti-GFP antibody. Precipitates were analyzed by western blotting using anti-GFP, or anti-Spo15 antibody.
(TIF)

S1 Movie Behavior of GFP-Psy1 and mCherry-Atb2 in wild type. The movie essentially corresponds to the images shown in Fig 1C. The movie plays at 10 frames per second.
(AVI)

S2 Movie Behavior of GFP-Psy1 and mCherry-Atb2 in *dms1Δ*. The movie essentially corresponds to the images shown in Fig 1C. The movie plays at 10 frames per second.
(AVI)
