# Supplementary material for: The fission yeast SPB component Dms1 is required to initiate forespore membrane formation and maintain meiotic SPB components
Source: PLoS One. 2018 May 29;13(5):e0197879. doi: 10.1371/journal.pone.0197879 (PMC5973557; doi:10.1371/journal.pone.0197879)
Supplement: S1 Text — (DOCX) [file pone.0197879.s001.docx]

To assess the interaction between Dms1 and Spo15 *in vivo*, wild-type cells harboring pREP41(GST) (CAL114) or pREP41(GST-dms1) (CAL115) were cultured in liquid minimal medium (MM). Next, the cells were suspended in extraction buffer (50 mM Tris-HCl, pH 7.5, 10 mM EDTA, 2 mM EGTA, 200 mM NaCl, 1% Triton X-100, 1 mM PMSF, protease inhibitor cocktail (nacalai tesque, Kyoto, Japan) and ruptured with glass beads. The lysate was centrifuged at 17,800 x g for 15 min, and an aliquot of supernatant was incubated with glutathione Sepharose (GE Healthcare) for 1 hour at 4˚C to pull down Dms1. The solution was then centrifuged, and the pellet was suspended in sample buffer. Samples were electrophoresed on SDS-polyacrylamide gels, and western blot analysis was performed using a mouse anti-GFP antibody (Roche Diagnostics) and a rabbit anti-Spo15 antibody [18].
