# Supplementary figures and images for: The fission yeast SPB component Dms1 is required to initiate forespore membrane formation and maintain meiotic SPB components
Source: PLoS One. 2018 May 29;13(5):e0197879. doi: 10.1371/journal.pone.0197879 (PMC5973557; doi:10.1371/journal.pone.0197879)

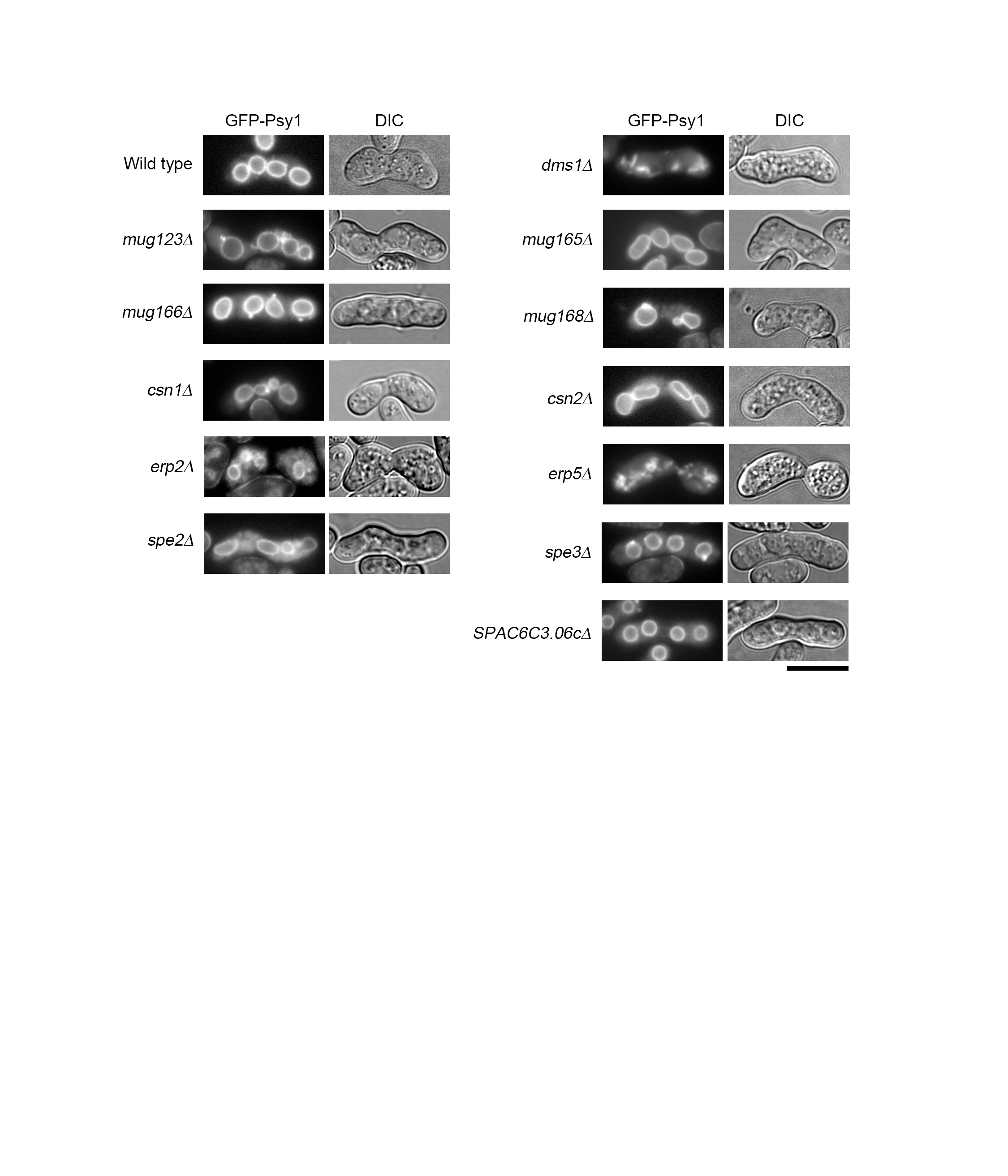

Supplement: S1 Fig — Homothallic haploid wild-type (KI36), csn1Δ (CAL130), csn2Δ (CAL8), erp2Δ (CAL39), erp5Δ (CAL45), mug123Δ (CAL56), mug165Δ (CAL27), mug166Δ (CAL31), mug168Δ (CAL124), SPAC6C3.06cΔ (CAL6), spe2Δ (CAL50), spe3Δ (CAL129), dms1Δ (CAL53), and tpp1Δ (CAL23) cells expressing GFP-Psy1 were sporulated on MEA for 16 hours and analyzed by fluorescence microscopy. Bar, 10 μm. (TIF) [file pone.0197879.s002.tif]

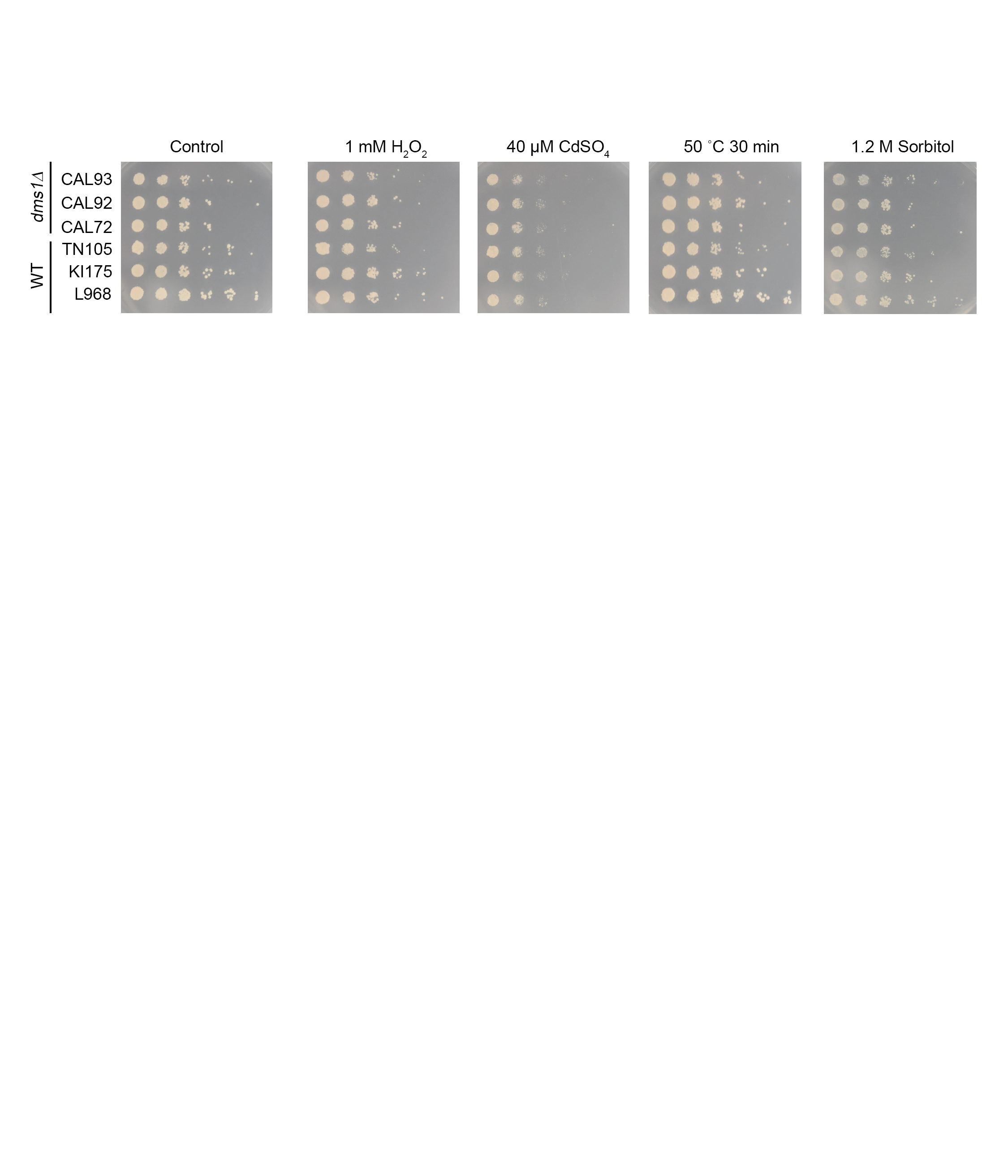

Supplement: S2 Fig — Homothallic haploid wild-type (L968, KI175, and TN105) and dms1Δ (CAL72, CAL92, and CAL93) strains were serially diluted from 102 to 107 cells/ml, spotted onto YEA, YEA + 1mM H2O2, YEA + 40 μM CdSO4 or YEA + 1.2 M sorbitol plates, and incubated at 30°C for 2 days. To test heat shock stress, cells were spotted onto YEA and treated at 50°C for 30 min before incubation at 30°C for 2 days. (TIF) [file pone.0197879.s003.tif]

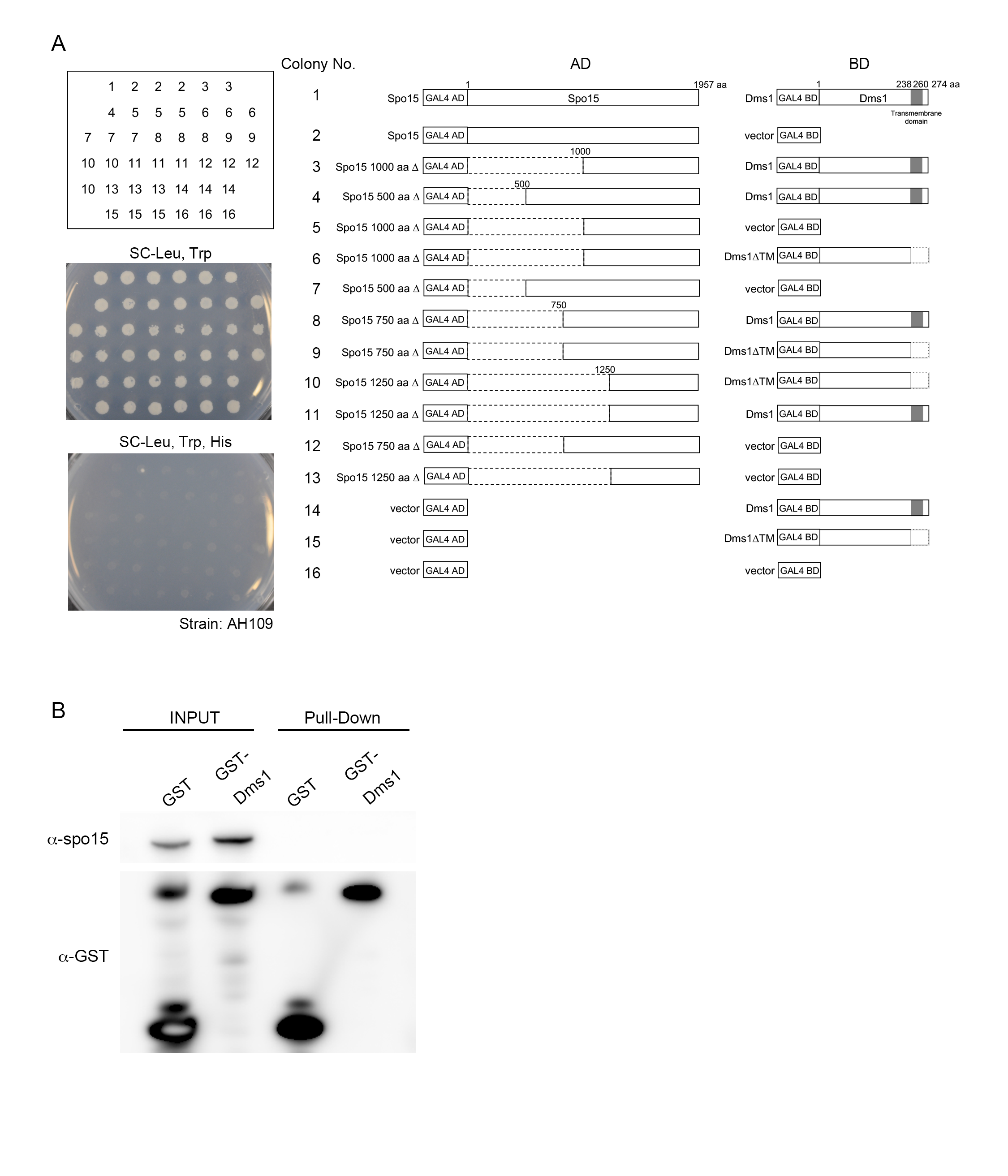

Supplement: S3 Fig — (A) Yeast two-hybrid analysis. Plasmids expressing the respective Gal4 activation domain (AD) and Gal4 DNA-binding domain (BD) fusions were tested for two-hybrid interaction. (B) Pull-down assay. Cell extracts were prepared from vegetative cells expressing the tagged proteins, GST or GST-Dms1, were subjected to pull down with anti-GFP antibody. Precipitates were analyzed by western blotting using anti-GFP, or anti-Spo15 antibody. (TIF) [file pone.0197879.s004.tif]
